# Supplementary figures and images for: A neural network model for the orbitofrontal cortex and task space acquisition during reinforcement learning
Source: PLoS Comput Biol. 2018 Jan 4;14(1):e1005925. doi: 10.1371/journal.pcbi.1005925 (PMC5771635; doi:10.1371/journal.pcbi.1005925)

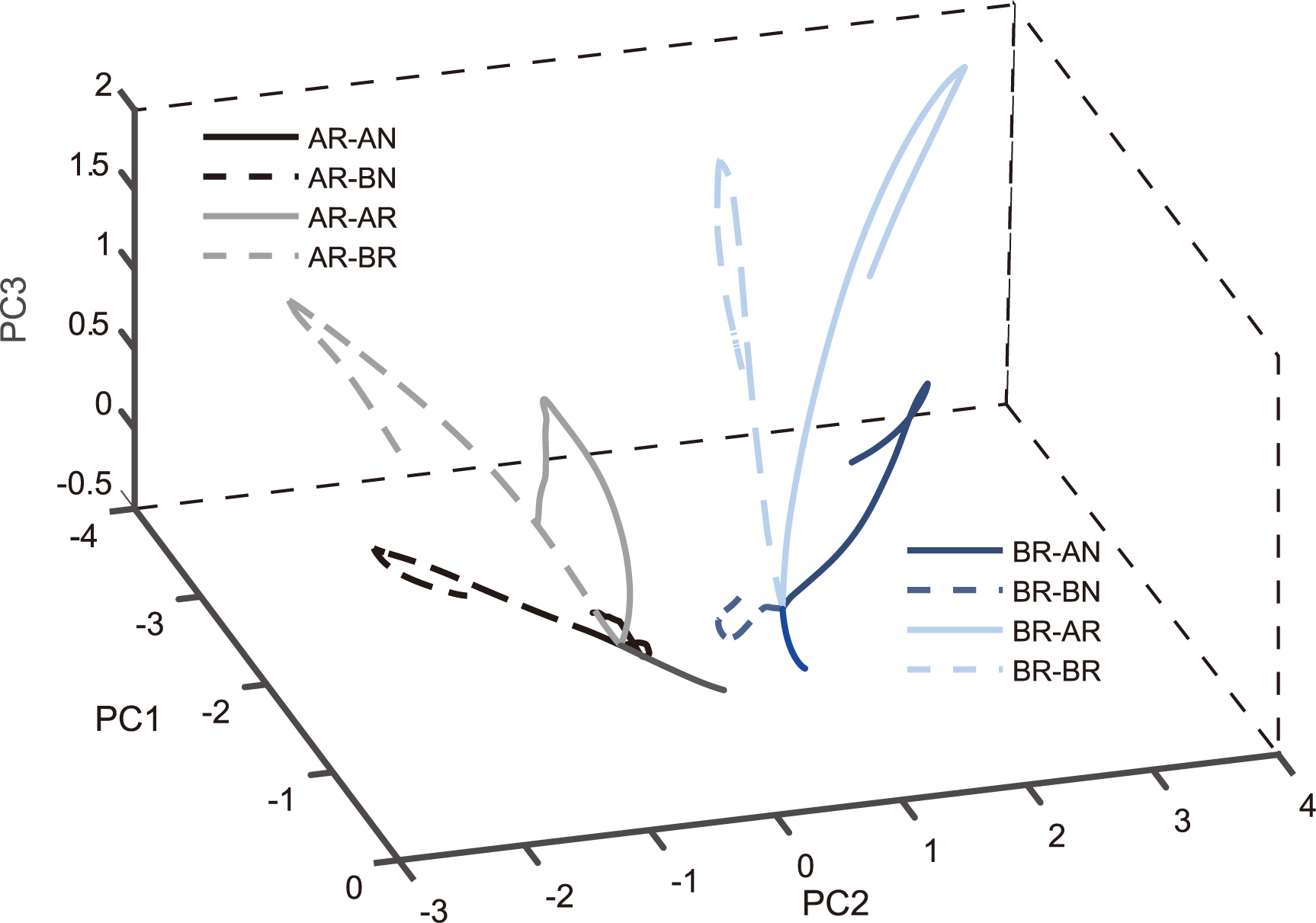

Supplement: S1 Fig — The sub-networks AR and BR consist of neurons that are selective to AR and BR, respectively. The network states are plotted in the space spanned by the first 3 PCA components, which are from the same PC space as in Fig 2B and are calculated from all neurons in the network. Each trace represents a different stimulus condition. (TIF) [file pcbi.1005925.s001.tif]

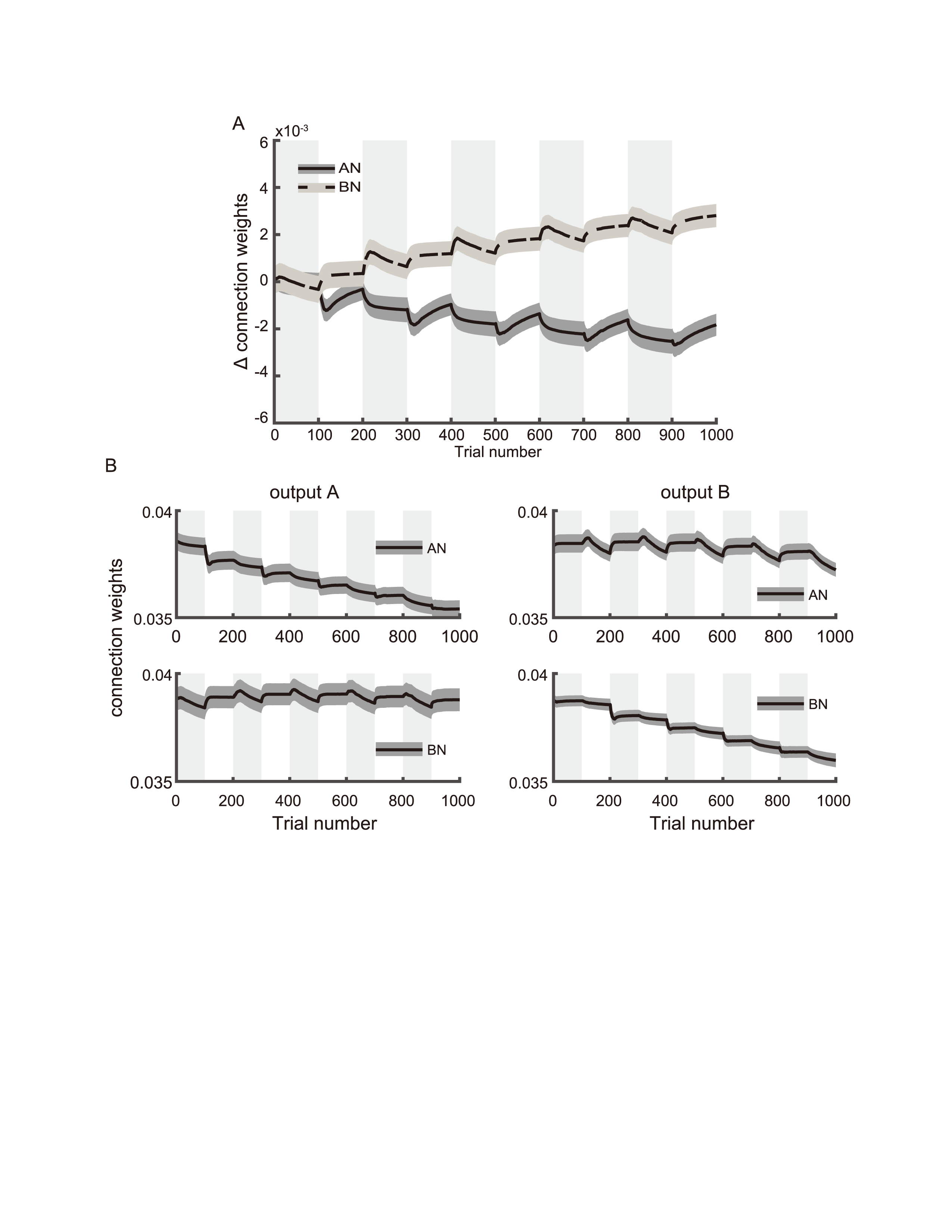

Supplement: S2 Fig — A. The difference between the connection weights of the AN and BN neurons in the SEL layer to DML unit A and DML unit B. Positive values indicate an SEL neuron has a stronger connection to DML unit A than to DML unit B and supports choice A. The gray and white area indicates the blocks in which the option A and the option B leads to the reward, respectively. B. Top row: AN neurons’ connections weight to DML unit A (left) and DML unit B (right). Bottom row: BN neurons’ connections weight to DML unit A (left) and DML unit B (right). (TIF) [file pcbi.1005925.s002.tif]

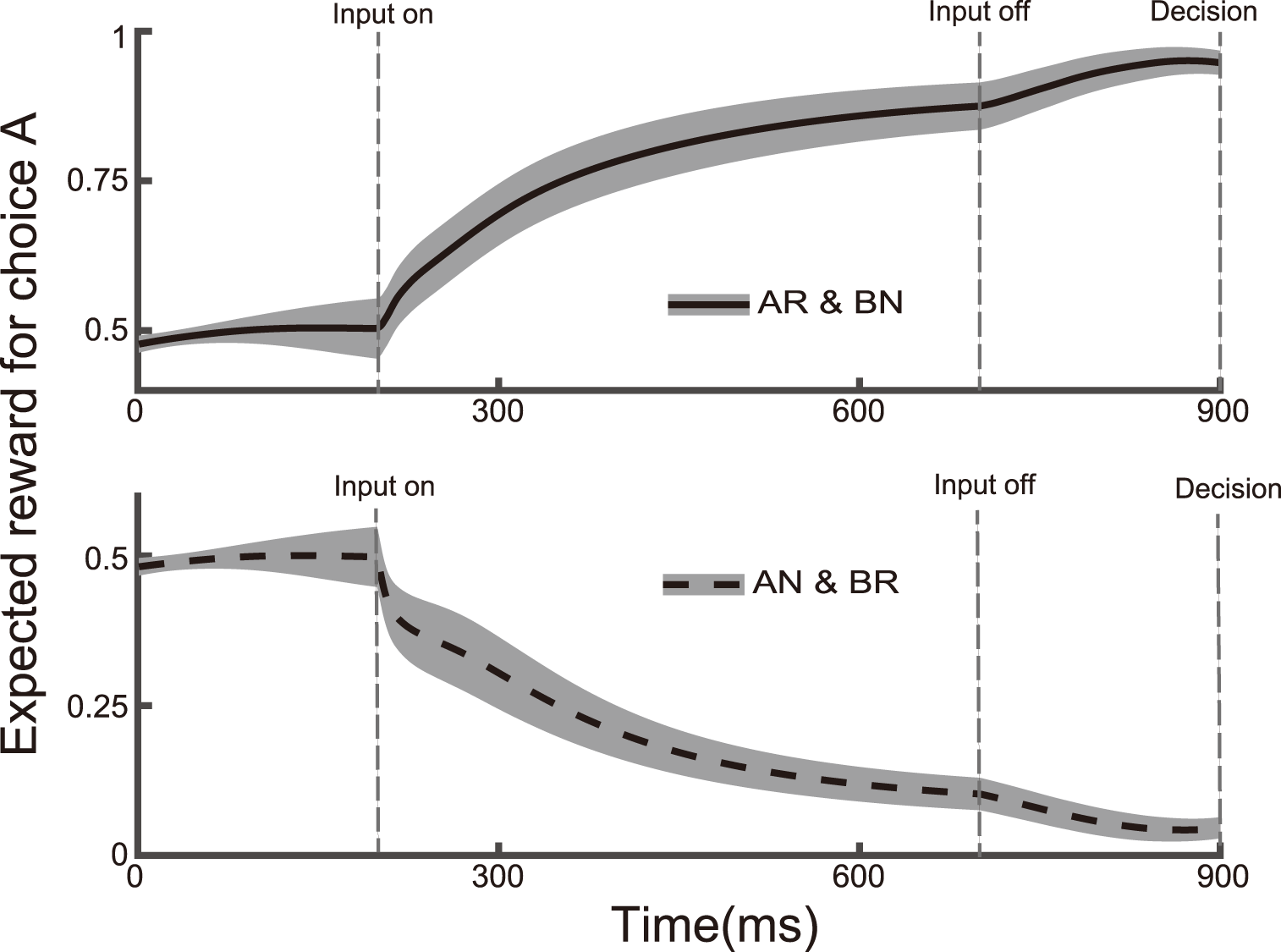

Supplement: S3 Fig — Shade area indicates the standard s.e.m. E[r] at 900 ms (decision time) is used for updating the weights. (TIF) [file pcbi.1005925.s003.tif]

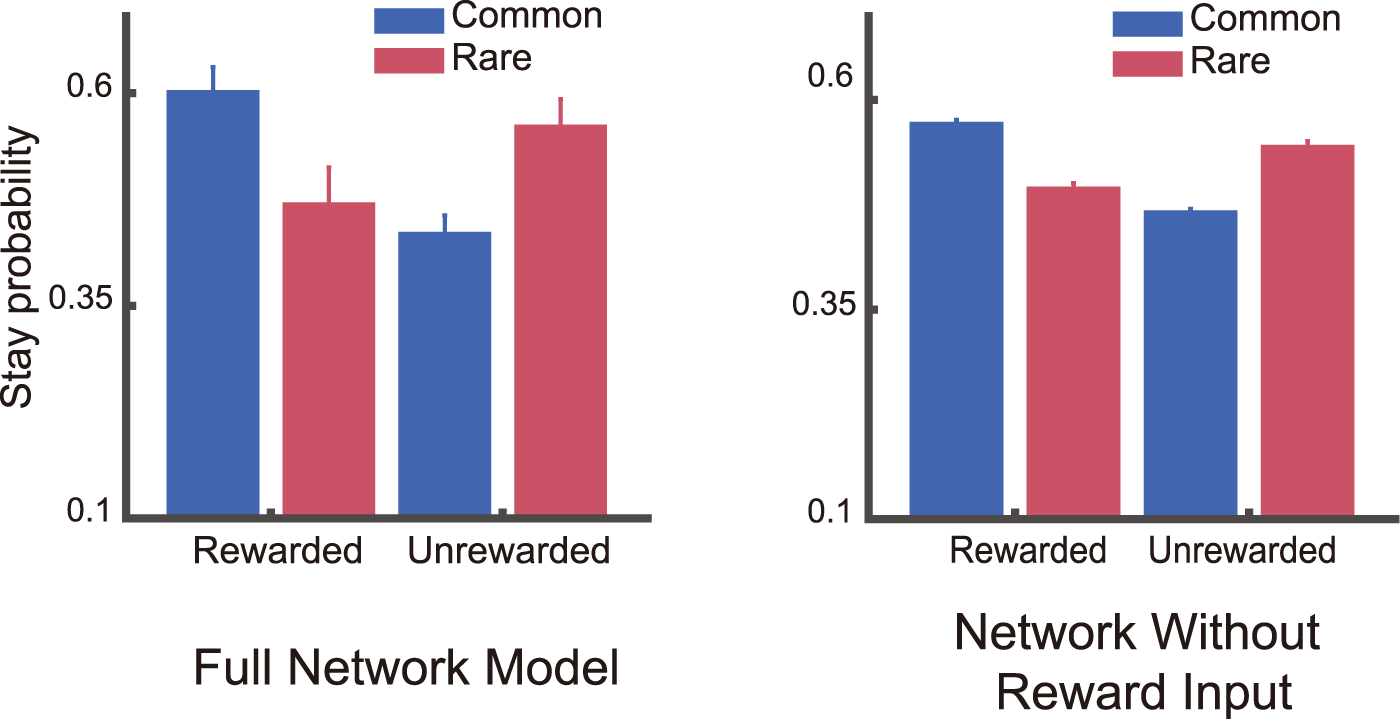

Supplement: S4 Fig — The full model exhibits stronger task-structure effects. (TIF) [file pcbi.1005925.s004.tif]

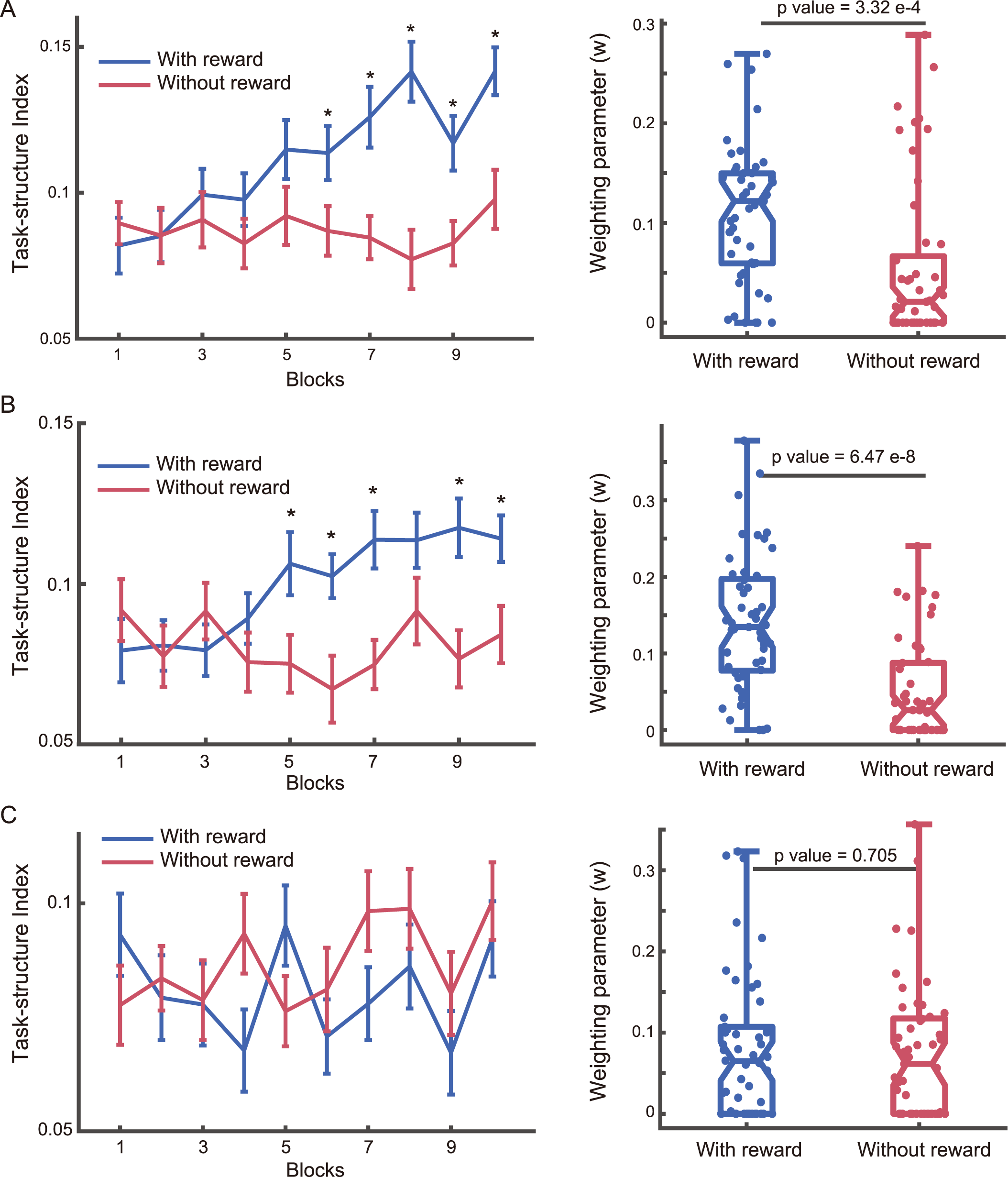

Supplement: S5 Fig — A. tau = 100 ms, A1/A2 occurs first at 200ms after the trial onset, and B1/B2 occurs at 700ms after the trial onset. B. tau = 500 ms, B1/B2 occurs first at 200ms after the trial onset, and A1/A2 occurs at 700ms after the trial onset. C. tau = 100 ms, B1/B2 occurs first at 200ms after the trial onset, and A1/A2 occurs at 700ms after the trial onset. Left column: the model structure index. Right column: weights for the model-based behavior. All the significance is evaluated by one-way ANOVA. See Fig 4B and 4C for details. (TIF) [file pcbi.1005925.s005.tif]

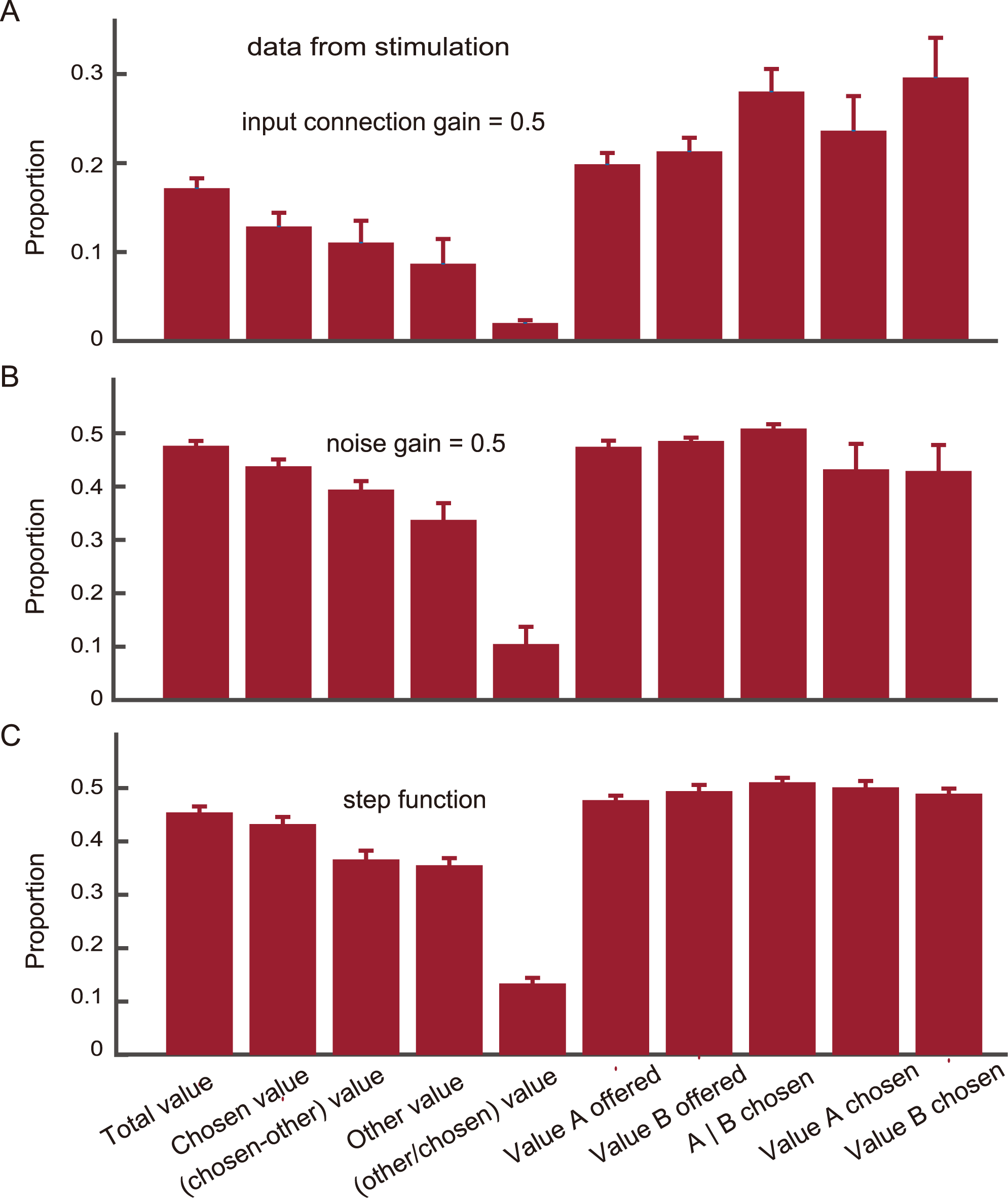

Supplement: S6 Fig — A. input connection gain = 0.5. B. noise gain = 0.5. C. A step function is used to model the reward inputs. The step function’s onset is 300 ms and its offset is 1300 ms after the trial onset. All results are based on 10 simulation runs. (TIF) [file pcbi.1005925.s006.tif]
